# ProAll-D: protein allergen detection using long short term memory - a deep learning approach

**DOI:** 10.5599/admet.1335

**Published:** 2022-09-13

**Authors:** Pallavi M. Shanthappa, Rakshitha Kumar

**Affiliations:** Department of Computer Science, Amrita School of Arts and Sciences, Mysuru Campus, Amrita Vishwa Vidyapeetham, India

**Keywords:** Allergen prediction, ACC transformation, LSTM model, Gaussian naive bayes, Classifier, Extra tree classifier, Bagging classifier, ADA boost, Linear discriminant analysis, Quadratic discriminant analysis

## Abstract

**Background:**

An allergic reaction is the immune system's overreacting to a previously encountered, typically benign molecule, frequently a protein. Allergy reactions can result in rashes, itching, mucous membrane swelling, asthma, coughing, and other bizarre symptoms. To anticipate allergies, a wide range of principles and methods have been applied in bioinformatics. The sequence similarity approach's positive predictive value is very low and ineffective for methods based on FAO/WHO criteria, making it difficult to predict possible allergens.

**Method:**

This work advocated the use of a deep learning model LSTM (Long Short-Term Memory) to overcome the limitations of traditional approaches and machine learning lower performance models in predicting the allergenicity of dietary proteins. A total of 2,427 allergens and 2,427 non-allergens, from a variety of sources, including the Central Science Laboratory and the NCBI are used. The data was divided 80:20 for training and testing purposes. These techniques have all been implemented in Python. To describe the protein sequences of allergens and non-allergens, five E-descriptors were used. E1 (hydrophilic character of peptides), E2 (length), E3(propensity to form helices), E4(abundance and dispersion), and E5 (propensity of beta strands) are used to make the variable-length protein sequence to uniform length using ACC transformation. A total of eight machine learning techniques have been taken into consideration.

**Results:**

The Gaussian Naive Bayes as accuracy of 64.14 %, Radius Neighbour's Classifier with 49.2 %, Bagging Classifier was 85.8 %, ADA Boost was 76.9 %, Linear Discriminant Analysis has 76.13 %, Quadratic Discriminant Analysis was 84.2 %, Extra Tree Classifier was 90%, and LSTM is 91.5 %.

**Conclusion:**

As the LSTM, has an AUC value of 91.5 % is regarded best in predicting allergens. A web server called ProAll-D has been created that successfully identifies novel allergens using the LSTM approach. Users can use the link https://doi.org/10.17632/tjmt97xpjf.1 to access the ProAll-D server and data.

## Introduction

Allergy, often described as an autoimmune disorder, is a clinical condition characterized by the immune system’s sensitivity to normally innocuous elements. The substance that causes allergy is known as an allergen. Allergens can be dust, pollen, cosmetics, and food. In food, allergy is usually caused by proteins. Proteins are an essential part of our diet, but some proteins can also be harmful to some individuals. One of the reasons behind this is that nowadays, the use of genetically modified crops that are transgenic food crops is increasing rapidly. Thus, it is necessary to assess them before they are introduced into the food chain. Allergy can be innate, acquired, predictable, and at times rapid. Allergic reactions are caused by an antibody called immunoglobulin E (IgE), which causes hyperactivity in white blood cells such as mast cells and basophils, resulting in the production of inflammatory chemicals like histamine. Apart from symptoms such as uneasiness, sneezing, wheezing, and swelling, allergic reactions can also lead to life-threatening situations. As a result, assessing them is critical to protect society's wellbeing.

According to the Food and Agriculture Organization, a protein is a potential allergen if it has a homology of six successive amino acids or a sequence identity of more than 35 percent [1]. Poms et al. developed PCR (Polymerase Chain Reaction) in conjunction with ELISA (Enzyme-Linked Immunosorbent Assay) to find potential allergens in foods or an indicator to detect the existence of the offending foods [2]. AlgPred uses MEME/MAST motif search to predict allergens and SVM for classification based on single and dipeptide composition [3]. AllerHunter uses SVM as the classifying method and an incremental pairwise sequence comparison indexing approach to identify probable allergens and allergic cross-reactivity in proteins. The paired vectorization system models the essential elements of allergens that are involved in cross-reactivity [4]. Using Pseudo-Amino Acid Composition (PseAAC) and SVM, a new technique for identifying and predicting allergenic proteins was developed. It looked at sequence vector representations derived from sequence attributes. The minimum Reliability and Maximal Significance feature selection approach were used to assess the impact and efficiency of each feature [5]. Vijaykumar et al. developed an innovative fuzzy rule-based approach to investigate protein allergenicity when the similarity between known allergens and non-allergens is low for characterizing allergens. The results of five different modules were combined: computational classifier, pattern analysis, global comparison with allergens, FAO management framework, and prototype approach [6].

AllerTop was developed as an alignment-free allergen prediction method. Protein properties were defined using Z-descriptors. The ACC transformation was used to convert the variable-length strings to uniform-length strings. The KNN (K-Nearest Neighbor) algorithm was applied for classification and consistently outperformed other algorithms [7]. AllergenFP- was designed for distinguishing allergens and non-allergens, and a sequence descriptor-based fingerprint technology was presented. The strings of varying lengths were transformed into arrays of similar lengths using the ACC transformation. The results were compared using Tanimoto coefficients followed by the transformation of vectors to binary fingerprints [8]. For allergenicity prediction, Dimitrov et al. developed Artificial Neural Network-based algorithms. As a final step before the ANN modeling, the vectors were transformed into binary fingerprints [9]. AllerTop v2 is a highly accurate allergen prediction model based on amino acid characteristics. The ACC procedure was used to transform variable-length strings into uniform-length vectors. In comparison to other classification approaches, the KNN algorithm produced a stable output graph [10]. Allerdictor is a pattern-based allergy prediction software that interprets sequence data as a textual information and detects allergens through text classification using support vector machines [11]. Cross-React was a computational framework approach for predicting allergenic protein’s cross-reactivity. It is based on the hypothesis that surface regions with peptide compositions similar to an antigen in a known allergen can be detected on three-dimensional structures of probable allergens [12].

A study of GE (Genetically Engineered) crops found they are as safe as conventional food crops. Screening the recombinant protein for predicting potential allergens is one of the assessment procedures for GE crops. It implies that there is currently no clear parameter that can be used to anticipate the pathogenicity of proteins [13]. AllerCatPro analyses potential allergenic protein based on the three-dimensional structural similarity. Shifting between sequential frame similarities to B-cell epitope-like 3D surface similarity with anticipated architectures was investigated, and so an entropy-adjusted hexamer hit method was also investigated [14]. Pallavi et al. used computational analysis to compare three medications and identified the impediment to malignant cells that cause skin cancer. They also used homology modeling to create the 3D framework of a BRAF(V600E) protein genotype, which they confirmed using the Ramachandran plot [15]. Aller Screener predicts protein allergenicity by analyzing HLA binders derived from recognized allergens. By creating binders to HLA class II proteins, it could predict whether a given substance is safe to eat or drink [16]. AlgPred v 2.0 provides various options, including searching for motifs in proteins found by MEME/MAST and MERCI features such as BLAST-based similarity searches and IgE epitope mapping [17]. Wang et al. demonstrated the superiority of their proposed technique by using numerous supervised algorithms as baseline classifiers. The greatest AUC value was 0.9578 for the deep learning model, which was superior to the ensemble learning and baseline approaches, according to the results of 5-fold cross-validation [18].

This paper comprises the report on the development methods of a set of novel allergen prediction models that use the knowledge gained via a publicly available server: AllerTOP v.2 [10]. We propose the Long Short-Term Memory (LSTM) as a rapid model-based recurrent neural network for protein allergen detection. LSTM is primarily used to handle long-term dependence problems and is best suited for time series or sequential data. Because our dataset, the protein dataset, is similarly in sequential form, LSTM would be a better method for classification with enhanced performance. Also, a few machine learning and ensemble learning algorithms have been evaluated for comparison and classification purposes.

## Methods

### Protein data sets

**Figure 1.**
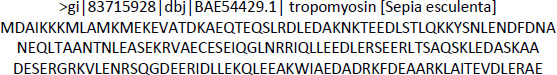
Sample dataset in Fasta Format of a protein sequence

We gathered a total of 2,427 allergens and 2,427 non-allergens from a variety of sources, including the Central Science Laboratory and the National Center for Biotechnology Information (NCBI). The redundancies were eliminated. A sample protein sequence is shown in Figure 1.

### E-descriptors

Five E-descriptors were used to characterize the protein sequences of allergens and non-allergens [10]. Venkatarajan et al. used principal component analysis to calculate the quantitative descriptor values based on 237 physical-chemical properties of amino acids. PCA of amino acid properties extracted 5 orthogonal E-descriptors, which are the eigenvectors of the covariance matrix [19]. We have considered the same five E-descriptors that were used to define the characteristics of amino acids [10]. E1 denotes the hydrophilic nature of peptides, E2 their length, E3 their tendency for helical formation, E4 their abundance and distribution, and E5 their tendency for β strand formation.

### Auto cross-covariance transformation

Proteins are composed of amino acid sequences, each distinct and varies in length. So, ACC transformation was employed to convert the variable-length sequence to uniform length so that the classification algorithms could be applied to it. Here, the 5 E-Descriptors have been considered, which were derived from 237 physio-chemical properties of amino acids as used by Dimitrov et al. [10].

Auto Cross Covariance includes Auto Covariance and Cross Covariance. The equation to calculate Auto covariance is as follows:


(1)

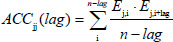



The equation to calculate Cross covariance is as follows:


(2)

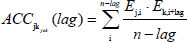



Index *j* was used for the *E-descriptors* (*j* = 1, 2, 3, 4, 5), *n* is the number of amino acids in a sequence, the index *i* is the amino acid position (*i* = 1, 2, ...n) and *l* is the lag, length of the minimum sequence (*l* = 1, 2, ...*L*). In order to investigate the influence of close amino acid proximity on protein allergenicity, a short range of lags (*L* = 1, 2, 3, 4, 5) was used [20].

### The classification methods adopted in this research

For classification, a few machine learning, ensemble learning, and deep learning algorithms have been considered, which include: The Gaussian Naive Bayes, Radius Neighbour's Classifier, Bagging Classifier, ADA Boost, Linear Discriminant Analysis, Quadratic Discriminant Analysis, Extra Tree Classifier, and LSTM, which have been implemented in Python.

Hochreiter and Schmidhuber proposed the term LSTM (Long Short-Term Memory) in 1997. LSTMs are a kind of RNN (Recurrent Neural Networks) that aid in the resolution of the long-term dependence problem. A conventional RNN encounters the difficulty of vanishing gradient problems, which makes learning extended sequences difficult. This is where LSTM comes in to address the challenge described above. The LSTM model is built iteratively. To construct the model, one must add different sorts of layers with varied parameters and experiment with dropout layers. The network constructed here consists of four layers with Three relu activation functions and one SoftMax function. "Categorical cross-entropy," was considered as the loss function with "rmsprop" as the optimizer.

### Evaluation of performance

For training and testing purposes, the data was split in the ratio 80:20. The accuracy results of the model based on training and testing data has been represented in Table 2. True positives (TP) and true negatives (TN) were assigned to the allergens and non-allergens that were accurately predicted. False negatives (FN) and false positives (FP) were assigned to the allergen and non-allergen identified inaccurately. Precision [(TP) /(TP + FP)] is the fraction of correctly predicted samples among the retrieved instances. The Recall [(TP)/(TP + FN)] is the ratio of accurately identified true positives to total actual true positives. And the F1 score is computed as [(2 *(Accuracy * Recall)/(Precision + Recall)].

### Web server for allergenicity prediction

A web server, namely ProAll-D, has been developed to predict the potential allergens using the LSTM algorithm. It is developed using the Python Django framework, which is fast and user-friendly. The detailed functioning of the webserver has been described in the supplementary section.

## Results and discussion

**Figure 2.**
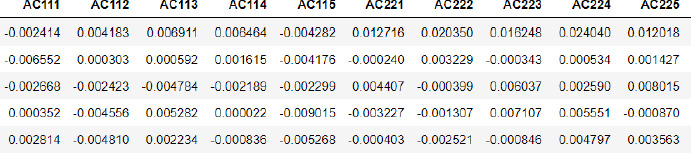
Output of ACC transformation

ACC includes both autocovariance and cross-covariance. Auto covariance is calculated between the same E Descriptors, that is between E1 and E1, along with the lag value. AC111 represents the autocovariance between E1 and E1 along with the lag value as shown in Figure 2. Cross covariance is calculated between the different E Descriptor values, like between E1 and E2, along with the lag value. The cross-covariance values will be represented as AC121, AC131, AC145, and AC431. The details of ACC implementation have been specified in the supplementary data.

The classification methods considered in this research are: The Gaussian Naive Bayes, Radius Neighbour's Classifier, Bagging Classifier, ADA Boost, Linear Discriminant Analysis, Quadratic Discriminant Analysis, Extra Tree Classifier, and LSTM, which have been implemented in Python.

Gaussian Naive Bayes is a statistical predictive model for classification that is based on the Naive Bayes algorithm. For our dataset, this algorithm produced an accuracy of 64.14 percent (Table 1). The Radius Neighbours Classifier is an extended version of the KNN algorithm that produces results using all instances within a range of a new instance rather than the k clusters, which would be beneficial for our dataset, but the model failed to provide the expected results, resulting in an accuracy of 49.2 percent. AdaBoost, also known as Adaptive Boosting, is an Ensemble Method. The weights are reallocated to each instance, with larger weights applied to inaccurately identified instances. Boosting is used in supervised learning to minimize bias as well as variation. The accuracy of this model was 76.9 percent.

Linear and Quadratic Discriminant Analysis resulted in an accuracy of 76.13 and 84.2 percent. A Bagging classifier is an ensemble classifier that works on random samples of the data and then combines various instances to obtain the final output. This method resulted in an accuracy of 85.8 percent. Extra tree classifier, an extended version of the random forest algorithm, resulted in an accuracy of 90 percent. LSTM model resulted in an accuracy of 91.5 percent.

Table 1 represents the results of performance evaluation metrics and accuracy of all the algorithms implemented. It is evident that the LSTM approach is superior and has consistent performance across all measures. So, LSTM has been considered for protein allergen prediction.

Table 2 represents the accuracy of the algorithms for training and testing dataset. The LSTM method was implemented to a well-known benchmark data set for protein allergen identification, in which a protein has to be classified as allergen or non-allergen. LSTM delivers highly defined classification performance that is substantially quicker than other algorithms with comparable classification performance. LSTM is five times faster than marginal classification algorithms (methods based on distance) and two times faster than the quickest SVM-based methods (which have lower classification performance than LSTM). All the implemented methods were tested and compared using performance evaluation measures. The top-performing model was LSTM, which had an accuracy of 91.51 percent. LSTM is a more sophisticated version of the RNN (recurrent neural network). The LSTM has been considered for our problem because of its robustness against long-term dependency problems. Since the protein sequences are also correlated with each other, LSTM would be a likely method for solving long-term dependencies and would overcome the drawbacks of the alignment method.

In Table 3 the performance of the LSTM model was compared to nine freely available servers. The LSTM resulted in an accuracy of 91.5 percent.

A ROC curve (short for receiver operating characteristic) plots the rate of true positives vs. false positives to assess the effectiveness of a classification model. AUC measures how well a model distinguishes between positive and negative classes. AUC is not affected by the classification threshold value. Modifying the threshold value does not affect AUC because it is an aggregate measure of ROC.

**Figure 3.**
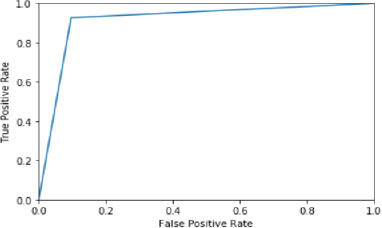
The LSTM model’s ROC curve

Figure 3 indicates that when the true positive rate increases, so does the false positive rate and after a certain point that is near 0.91 the graph is constant. The area under the ROC curve between (0,0) and (1,1) is defined as the area under the curve (AUC). AUC essentially aggregates the model's performance overall threshold values.

The LSTM model has the highest AUC, indicating that it has the largest area under the curve and is the best model for correctly classifying observations. The software for the server ProAll-D has been developed to predict potential allergens using the LSTM algorithm. It is developed using the Python Django framework, which is fast and user-friendly.

**Figure 4.**
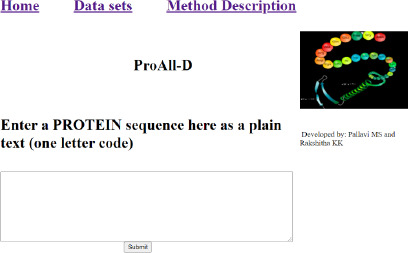
Interface of ProAll-D for protein allergen detection.

**Figure 5.**
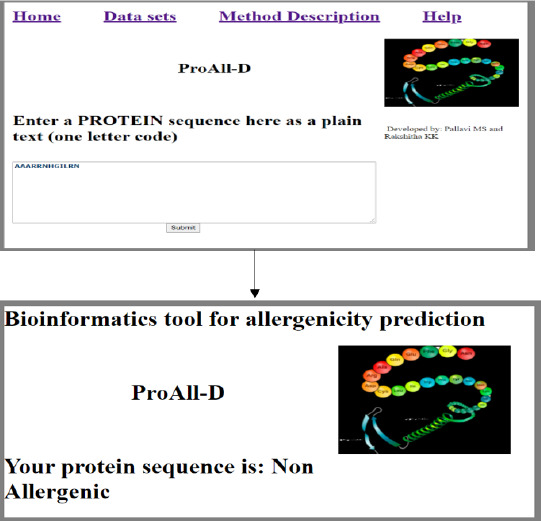
Working of ProAll-D.

**Figure 6.**
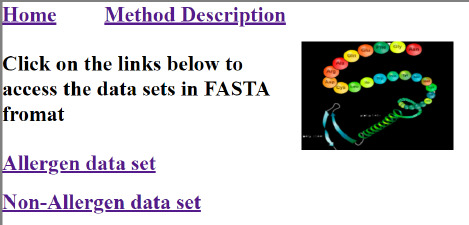
Data-set Section

There are three different sections, namely Home, Datasets, and Method Description as shown in Figure 4. In the home section, the user enters the protein sequence in a one-letter code, and the models predict whether the entered sequence is allergenic or non-allergenic as shown in Figure 5. In the dataset part, we have uploaded the data considered in our research in the Fasta file format as represented in Figure 6. The Method description provides the user with a brief description of the methodologies we have considered.

The supplementary section describes the detailed functioning of the web application.

## Conclusions

This study builds on and expands earlier studies to develop the analysis of potential protein allergens. Our first aim has been to update the technique while keeping the previous factors in mind. We evaluated deep learning, ensemble learning, and machine learning models such as the Gaussian Naive Bayes, Radius Neighbour’s Classifier, Bagging Classifier, ADA Boost, Linear Discriminant Analysis, Quadratic Discriminant Analysis, and LSTM to predict the allergenicity of proteins. Extensive testing produced excellent results. They were superior and corroborated earlier research. Furthermore, the AUC value of LSTM (the best performance) was 0.9152. So far, this is the only study to apply the aforementioned methods to evaluate protein allergenicity, and it will serve as a paradigm for future protein allergen prediction.



## Figures and Tables

**Table 1.** Analysis of the effectiveness of different classifiers

Method	Accuracy	Precision	Recall	F1-Score
Gaussian Naive Bayes	64.14	0.74	0.46	0.56
Radius Neighbours Classifier	49.2	0.49	1.00	0.66
ADA Boost	76.9	0.79	0.75	0.77
Linear Discriminant Analysis	76.13	0.80	0.71	0.75
Quadratic Discriminant Analysis	84.2	0.93	0.74	0.83
Bagging Classifier	85.8	0.89	0.86	0.88
Extra Tree Classifier	90	0.95	0.85	0.90
LSTM (Long Short-Term Memory)	91.5	0.91	0.91	0.91

**Table 2.** Analysis of the classification results of different classifiers based on training and testing data.

Method	Training data	Testing data
Gaussian Naive Bayes	63.8	59.7
Radius Neighbours Classifier	50.5	47.6
ADA Boost	84.6	78.1
Linear Discriminant Analysis	78.6	74.9
Quadratic Discriminant Analysis	88.7	81.6
Bagging Classifier	88.4	86.3
Extra Tree Classifier	93.4	89.8
LSTM (Long Short-Term Memory)	94.1	91.5

**Table 3.** Assessment of web servers for allergenicity prediction

Server	Accuracy
Allerhunter	0.871
AlgPred (SVM_single_aa)	0.775
AlgPred (SVM_dipeptide)	0.796
AlgPred(ARP)	0.842
APPEL	0.783
ProAp(motif)	0.505
ProAp(SVM)	0.843
AllerTop v.1	0.828
AllergenFP	0.879
AllerTop v.2	0.887
LSTM model	0.915
