# Supplementary material for: ProAll-D: protein allergen detection using long short term memory - a deep learning approach
Source: ADMET DMPK. 2022 Sep 13;10(3):231–40. doi: 10.5599/admet.1335 (PMC9484702; doi:10.5599/admet.1335)
Supplement: Supplementary file 1 [file Admet-10-1335_S1.pdf]

Open Access : ISSN: 1848-7718

http://www.pub.iapchem.org/ojs/index.php/admet/index

Supplementary material

ProAll-D: Protein Allergen Detection using Long Short Term Memory- A Deep Learning Approach

Pallavi M S*, Rakshitha K K[1]

Department of Computer Science, Amrita School of Arts and Sciences, Mysuru Campus, Amrita Vishwa Vidyapeetham, India

*Corresponding Author: E-mail:palls.ms@gmail.com ;

SUPPLEMENTARY MATERIAL

https://doi.org/10.5599/admet.1335

Dataset Collection

The protein data have been collected from various sources including the: Central Science Laboratory and the National Center for Biotechnology Information (NCBI) The redundancies were eliminated.

The sources from which the data has been gathered are mentioned below:

[National Center for Biotechnology Information (nih.gov)](https://www.ncbi.nlm.nih.gov)

http:// www.ddg-pharmfac.net/AllergenFP/data.html.

```
>gi|75139847|sp|Q7M1E8|Q7M1E8_9ROSA Pollen major allergen Par o I
ATGKVVQGAMPP
>P0A0L2 RecName: Full=Enterotoxin type A; AltName: Full=SEA; Flags: Precursor;
MKKTAFTLLLFIALTLTTSPLVNGSEKSEEINEKDLRKKSELQGTALGNLKQIYYYNEKA
KTENKESHDQFLQHTILFKGFFTDHSWYNDLLVDFDSKDIVDKYKGKKVDLYGAYYGYQC
AGGTPNKTACMYGGVTLHDNNRLTEEKKVPINLWLDGKQNTVPLETVKTNKKNVTVQELD
LQARRYLQEKYNLYNSDVFDGKVQRGLIVFHTSTEPSVNYDLFGAQGQYSNTLLRIYRDN
KTINSENMHIDIYLYTS
>gi|83715928|dbj|BAE54429.1| tropomyosin [Sepia esculenta]
MDAIKKKMLAMKMEKEVATDKAEQTEQSLRDLEDAKNKTEEDLSTLQKKYSNLENDFDNA
NEQLTAANTNLEASEKRVAECESEIQGLNRRIQLLEEDLERSEERLTSAQSKLEDASKAA
DESERGRKVLENRSQGDEERIDLLEKQLEEAKWIAEDADRKFDEAARKLAITEVDLERAE
ARLEAAEAKIVELEEELKVVGNNMKSLEISEQEASQREDSYEETIRDLTHRLKEAENRAA
EAERTVSKLQKEVDRLEDELLAEKERYKSISDELDQTFAELAGY
>gi|131112|sp|P02622.1|PRVB_GADCA RecName: Full=Parvalbumin beta; AltName: Full=Allergen Gad c I; AltName: Full=Allergen M; AltName: Allergen=Gad c 1
AFKGILSNADIKAAEAACFKEGSFDEDGFYAKVGLDAFSADELKKLFKIADEDKEGFIEE
DELKLFLIAFAADLRALTDAETKAFLKAGDSDGDGKIGVDEFGALVDKWGAKG
>Q43552 SubName: Full=Major allergen Mal d1;
MGVFNYETEFTSVIPAPRLFKAFILDGDNLIPKIAPQAIKSTEIIEGDGGVGTIKKVTFG
EGSQYGYVKQRVNGIDKDNFTYSYSMIEGDTLSDKLEKITYETKLIASPDGGSIIKTTSH
YRAKGDVEIKEEHVKAGKEKASGLFKLLEAYLLAHSDAYN
>Q43550 SubName: Full=Major allergen Mal d 1;
MGVFNYETETTSVIPAPRLFKAFILDGDNLIPKIAPQAIKSTEIIEGDGGVGTIKKVTFG
EGSQYGYVKQRVNGIDKDNFTYSYSMIEGDTLSDKLEKITYETKLIASPDGGSIIKTTSH
YHAKGDVEIKEEHVKAGKEKASGLFKLLEAYLLAHSDAYN
>Q43551 SubName: Full=Major allergen Mal d1;
MGVFNYETEFTSVIPAPRLFKAFILDGDNLIPKIAPQAIKSTKIIEGDGGVGTIKKVTFG
EGSQYGYVKQRVNGIDKDNFTYSYSMIEGDTLSDKLEKITYETKLIASPDGGSIIKTNSH
YHAKGDVEIKEEHVKAGKEKASGLFKLLEAYLLAHSDAYN
>gi|76097507|gb|ABA39436.1| Der f 1 allergen precursor [Dermatophagoides farinae]
RPASIKTFEEFKKAFNKNYATVEEEEVARKNFLESLKYVEANKGAINHLSDLSLDEFKNR
YLMSAEAFEQLKTQFDLNAETSACRINSVNVPSELDLRSLRTVTPIRMQGGCGSCWAFSG
VAATESAYLAYRNTSLDLSEQELVDCASQHGCHGDTIPRGIEYIQQNGVVEERSYPYVAR
EQQCRRPNSQHYGISNYCQIYPPDVKQIREALTQTHTAIAVIIGIKDLRAFQHYDGRTII
QRDNGYQPNYHAVNIVGYGSTQGVDYWIVRNSWDTT
```

Fig 1. Raw data in fasta file format

The above figure represents the protein sequence in fasta file format. The symbol “>” indicates the data is in fasta format and it also specifies the beginning of the new sequences. The numerical present after the ‘>’ is the protein accession number and scientific name of the protein.

Since each sequence is present in multiple lines so it has to be converted to a single line. For this conversion, the Linux command has been used.

```
Terminal
~$ perl -pe'/^>/? print "\n" : chomp'Allergen(fasta_format).txt > single.txt
```

Fig 2. Linux command to convert multiple line sequence to a single line

```
>gi|71360930|emb|CAJ19706.1| non-specific lipid transfer protein [Solanum lycopersicum]
MEMFGKIACFVVFCMVVVAPHAESLSCGEVTSGLAPCLPYLEGRGPLGGCCGGVKGLLGAAKTPEDRKTACTCLKSAANSIKGIDTGKAAGLPGVCGVNIPYKISPSTDCSTVQ
>Q40239 SubName: Full=Allergen Lol p II; Flags: Fragment;
EFTVEKGSDEKNLALSIKYSKEGDAMAEVELKEHGSNEWLALKKNGDGVWEIKSDKPLKGPFNFRFVSEKGMWNVFDDVVPADFKVGT
```

Fig 3. Data converted from multiple lines to a single line

Methods

The E-descriptor values derived by Venkatarajan et al[23] has been considered to describe the features of proteins.

Amino acid	E1	E2	E3	E4	E5
Alanine (A)	0.008	0.134	−0.475	−0.039	0.181
Arginine (R)	0.171	−0.361	0.107	−0.258	−0.364
Asparagine (N)	0.255	0.038	0.117	0.118	−0.055
Aspartic acid (D)	0.303	−0.057	−0.014	0.225	0.156
Cysteine (C)	−0.132	0.174	0.07	0.565	−0.374
Glutamate (Q)	0.149	−0.184	0.03	0.035	−0.112
Glutamic acid (E)	0.221	−0.280	−0.315	0.157	0.303
Glycine (G)	0.218	0.562	−0.024	0.018	0.106
Histidine (H)	0.023	−0.177	0.041	0.28	−0.021
Isoleucine (I)	−0.353	0.071	−0.088	−0.195	−0.107
Leucine (L)	−0.267	0.018	−0.265	−0.274	0.206
Lysine (K)	0.243	−0.339	−0.044	−0.325	−0.027
Methionine (M)	−0.239	−0.141	−0.155	0.321	0.077
Phenylalanine (F)	−0.329	−0.023	0.072	−0.002	0.208
Proline (P)	0.173	0.286	0.407	−0.215	0.384
Serine (S)	0.199	0.238	−0.015	−0.068	−0.196
Threonine (T)	0.068	0.147	−0.015	−0.132	−0.274
Tryptophan (W)	−0.296	−0.186	0.389	0.083	0.297
Tyrosine (Y)	−0.141	−0.057	0.425	−0.096	−0.091
Valine (V)	−0.274	0.136	−0.187	−0.196	−0.299

ACC transformation

Auto Cross Covariance includes both Auto Covariance and Cross Covariance. Here the 5 E Descriptors has been considered and ACC transformation for converting amino acid sequence to a sequence of numbers so that we can apply classification algorithms to them

Let's take an example, we have a sequence as follows: ARN length = 3s

The respective E Descriptors for each amino acid are as follows:

Alanine (A) 0.008 0.134 −0.475 −0.039 0.181

Arginine (R) 0.171 −0.361 0.107 −0.258 −0.364

Asparagine (N) 0.255 0.038 0.117 0.118 −0.055

So, when this sequence is converted to an E Descriptor sequence we get: 0.008 0.134 −0.475 −0.039 0.181 0.171 −0.361 0.107 −0.258 −0.364 0.255 0.038 0.117 0.118 −0.055 length = 3*5=15

The ACC transformation on the E Descriptor sequence is done by using the respective formulas for autocovariance and cross-covariance. The autocovariance is calculated between the same E Descriptor, for eg auto covariance between E1 and E1 is represented as AC11, then we also incorporate the lag value, which in our case ranges from 1 to the length of minimum sequence i.e. from 1 to 5. so now finally:

The autocovariance between E1 and E1 and lag=1 will be represented as AC111

The autocovariance between E1 and E1 and lag=2 will be represented as AC112

The autocovariance between E2 and E2 and lag=1 will be represented as AC221, etc

Out[74]:

	AC111	AC112	AC113	AC114	AC115	AC221	AC222	AC223	AC224	AC225
**0**	-0.004641	0.003314	-0.010949	-0.002797	0.000705	0.004554	-0.012834	0.007513	0.003767	0.001959
**1**	0.002636	-0.001878	-0.003674	-0.005849	-0.002772	0.000733	0.003134	0.007477	0.002913	0.003250
**2**	-0.001600	-0.004875	-0.002582	-0.001582	0.000641	0.003093	0.004962	0.001689	0.002621	0.002371
**3**	-0.006489	-0.003626	0.003811	-0.007623	-0.000928	0.001987	0.000680	0.003707	0.001542	-0.000983
**4**	0.000686	-0.003400	0.001213	0.003140	0.003327	0.007952	0.002433	0.006808	0.000674	0.000139
**...**	...	...	...	...	...	...	...	...	...	...
**4415**	0.004306	0.004505	-0.004866	-0.007286	-0.001521	0.003876	-0.002050	-0.003842	0.005143	0.001283
**4416**	-0.003726	0.001502	0.000026	-0.006636	-0.001730	0.004304	0.001005	0.004774	0.002701	-0.000802
**4417**	0.016728	0.007909	-0.010960	-0.016533	-0.000428	0.012362	0.011170	0.010892	0.015810	0.007194
**4418**	-0.007640	0.000200	-0.005678	-0.004239	0.001960	-0.004110	0.000803	0.003362	0.012641	0.005196
**4419**	0.006893	0.008927	0.004723	0.009670	0.007001	-0.000942	0.000889	0.002527	0.006533	0.001714

AC224	...	AC532	AC533	AC534	AC535	AC541	AC542	AC543	AC544	AC545
0.003767	...	-0.003887	-0.002417	0.002399	-0.004025	-0.001182	-0.001535	-0.001253	0.000769	-0.011362
0.002913	...	0.001522	-0.001193	-0.002175	-0.002731	-0.000956	0.002548	0.000311	-0.000414	-0.002027
0.002621	...	0.001081	-0.004058	0.000364	-0.003639	-0.002833	-0.001185	-0.000779	-0.000066	-0.000732
0.001542	...	-0.004964	-0.002996	-0.003467	0.001623	-0.000086	-0.000436	-0.003409	0.001407	-0.001669
0.000674	...	0.003858	0.001022	0.000081	-0.003077	-0.003084	-0.001505	0.004391	0.003225	-0.001804

Fig 4 . Output of Autocovariance transformation

The same goes for cross-covariance, the only difference is that cross-covariance is calculated between different E Descriptor values, this means that the cross-covariance values will be represented as AC121, AC131, AC145, AC431, etc.

The autocovariance and cross-covariance values for each amino acid sequence are combined, then the sequence is said to be ACC transformed these ACC values are the attributes on the basis of which classification algorithms can be applied.


```
Header,AC111,AC112,AC113,AC114,AC115,AC221,AC222,AC223,AC224,AC225,AC331,AC332,AC333,AC334,AC335,AC441,AC442,AC443,AC444,AC445,AC551,AC552,
AC553,AC554,AC555,AC121,AC122,AC123,AC124,AC125,AC131,AC132,AC133,AC134,AC135,AC141,AC142,AC143,AC144,AC145,AC151,AC152,AC153,AC154,AC155,A
C211,AC212,AC213,AC214,AC215,AC231,AC232,AC233,AC234,AC235,AC241,AC242,AC243,AC244,AC245,AC251,AC252,AC253,AC254,AC255,AC311,AC312,AC313,AC
314,AC315,AC321,AC322,AC323,AC324,AC325,AC341,AC342,AC343,AC344,AC345,AC351,AC352,AC353,AC354,AC355,AC411,AC412,AC413,AC414,AC415,AC421,AC4
22,AC423,AC424,AC425,AC431,AC432,AC433,AC434,AC435,AC451,AC452,AC453,AC454,AC455,AC511,AC512,AC513,AC514,AC515,AC521,AC522,AC523,AC524,AC52
5,AC531,AC532,AC533,AC534,AC535,AC541,AC542,AC543,AC544,AC545,Class
>gi|86155962|gb|ABC86713.1| LEGCYC1B [Lupinus
angustifolius],0.0053845988372093,0.0023705730994152,-0.0011734882352941,0.002851573964497,0.002330619047619,-0.0001086686046511,0.00500233
33333333,0.0010791,-0.0006926804733727,0.0001848809523809,0.0015076162790697,0.0048904912280701,-0.0027734823529411,0.0041842840236686,-0.0
019199047619047,0.0058896337209302,0.0059418245614035,-0.0023388117647058,0.0024217751479289,0.004050363095238,-0.0057500697674418,0.005328
3450292397,0.0012268294117647,0.0028852958579881,0.000593494047619,0.0048660581395348,0.0012038421052631,0.00313194117647,0.00603803550295
85,0.0028732797619047,-0.0012740232558139,0.0001826257309941,-0.0019326352941176,0.0055900887573964,-0.0006766071428571,-0.0005620813953488
,0.0031249239766081,0.001690794117647,-0.0058329881656804,-0.0055094761904761,0.0048970116279069,-0.0023715380116959,-0.0019757176470588,-0
.0071116686390532,-0.0026896547619047,0.0021576162790697,-0.002297380116959,0.0004490999999999,-0.0007663136094674,-0.0033947916666666,0.00
98767616279069,-0.0038770409356725,0.0034447470588235,-0.0040521834319526,0.0032448095238095,-0.0063959476744185,0.0076889941520467,-0.0026
779176470588,0.0038892781065088,0.0015938511904761,-0.0052171860465116,0.0015222514619883,-0.0024262882352941,0.006163372781065,-0.00707836
9047619,-0.0031860174418604,-0.0020590584795321,-0.0061133529411764,0.001764597633136,-0.00441575,0.0058383139534883,-0.003868865497076,0.0
038647823529411,0.0077727396449704,0.0010454642857142,0.0058977558139534,0.0013924444444444,0.0051068,-0.0033013017751479,0.003431523809523
8,0.0072074941860465,-0.0022766608187134,0.0055243823529411,-0.0021535680473372,0.0095033214285714,-0.0035926453488372,-0.0033625263157894,
0.0002418764705882,0.0040067337278106,-0.0022484642857142,0.0046694476744186,0.0056278187134502,0.0075747411764705,0.0054752899408284,0.004
6454404761904,0.006055976744186,0.0005057192982456,0.0048279823529411,0.0049596686390532,0.0039706607142857,-0.0013822325581395,-0.00027738
59649122,-0.008393694117647,0.0022006923076923,0.00192075,0.002382203488372,-0.0031231578947368,-0.004326594117647,-0.0028009822485207,-0.0
005473988095238,-0.0084404709302325,0.0049344795321637,0.0078345,0.0026740769230769,0.0046146785714285,0.0037197965116279,0.003315678362573
,0.0032350647058823,-0.0043310946745562,0.000663113095238,0.0039959476744186,0.0076992046783625,-0.003820894117647,0.002502023668639,0.0005
18125,0
```


Fig 5. Output of autocovariance and cross covariance

The above snapshot indicates the header which contains the combined output of autocovariance and cross-covariance, there are 125 attributes based on which further the algorithms are applied.

Code for ACC Transformation

```
def represent_as_E_descriptors(allergens):
    allergens_E_descriptors = {}

    for key in allergens:
        allergens_E_descriptors[key] = []

    for key in allergens_E_descriptors:
        for base in allergens[key]:
            allergens_E_descriptors[key].extend(E_descriptors[base])
    return allergens_E_descriptors

def convert_to_2D(descriptors):
    E1 = []
    E2 = []
```

```
    E3 = []
    E4 = []
    E5 = []
    for i in range(0,len(descriptors),5):
        E1.append(descriptors[i])
        E2.append(descriptors[i+1])
        E3.append(descriptors[i+2])
        E4.append(descriptors[i+3])
        E5.append(descriptors[i+4])

    E = [E1,E2,E3,E4,E5]
    E = np.array(E)
    return E

def calculate_auto_covariance(j_list, lag, n, E):
    auto_covariance = {}
    for j in j_list:
        for l in lag:
            sum = 0
            for i in range(0,n-l):
                sum += (E[j,i] * E[j,i+l])/(n-l)
            key = 'AC'+str(j+1)+str(j+1)+str(l)
            auto_covariance[key] = sum
    return auto_covariance

def calculate_cross_covariance(j_list, k_list, lag, n, E):
    cross_covariance = {}
    for j in j_list:
        for k in k_list:
            if j == k:
                continue
            else:
                for l in lag:
                    sum = 0
                    for i in range(0,n-l):
                        sum += (E[j,i] * E[k,i+l])/(n-l)
                    key = 'AC'+str(j+1)+str(k+1)+str(l)
                    cross_covariance[key] = sum
    return cross_covariance

def main(input_file, output_file, Label, j_list, k_list, lag):
```

```
    allergens = {}
    allergen_df = pd.read_csv(input_file)

    headers = allergen_df[allergen_df.index % 2 == 0].apply(lambda x: x.str.strip('\t')).iloc[:,0]
    sequences = allergen_df[allergen_df.index % 2 != 0].apply(lambda x: x.str.strip('\t')).iloc[:,0]

    for header, sequence in zip(headers, sequences):
        allergens[header] = sequence

    allergens_E_descriptors = represent_as_E_descriptors(allergens)

    auto_covariance_df = pd.DataFrame()
    cross_covariance_df = pd.DataFrame()

    for key in allergens_E_descriptors:
        descriptors = allergens_E_descriptors[key]
        E = convert_to_2D(descriptors)
        n = len(descriptors)//5
        auto_covariance = calculate_auto_covariance(j_list, lag, n, E) # calling calculate_auto_covariance
fucntion
        tmp_1 = pd.DataFrame(auto_covariance, index = [0])
        auto_covariance_df = pd.concat([auto_covariance_df,tmp_1], axis = 'rows')

        cross_covariance = calculate_cross_covariance(j_list, k_list, lag, n, E) # calling
calculate_auto_covariance fucntion
        cross_covariance['Header'] = key
        tmp_2 = pd.DataFrame(cross_covariance, index = [0])
        cross_covariance_df = pd.concat([cross_covariance_df,tmp_2], axis = 'rows')

    final_df = pd.concat([auto_covariance_df,cross_covariance_df], axis = 'columns')
    final_df =final_df.reset_index(drop=True)

    first_column = final_df.pop('Header')
    final_df.insert(0, 'Header', first_column)
    final_df['Class'] = Label
    final_df.head()
    final_df.to_csv(output_file, index = False)

import pandas as pd
import numpy as np
```

```
allergen_file = 'allergens.csv'
non_allergen_file = 'NonAllergens.csv'
allergen_output_file = 'Allergen_ACC_transformed.csv'
non_allergen_output_file = 'Non_Allergen_ACC_transformed.csv'
allergen_label = 1
non_allergen_label = 0

# j_list is 0 to 4 because we have 5 E Descriptors
j_list = [0,1,2,3,4]

# lag is set to 5, as the in our data the length of shortest sequence is 5
lag = [1,2,3,4,5]

# we need this, exclusively for calculating cross covariance
k_list = [0,1,2,3,4]
E_descriptors = {'A': [0.008,0.134,-0.475,-0.039,0.181],
        'R': [0.171,-0.361,0.107,-0.258,-0.364],
        'N': [0.255,0.038,0.117,0.118,-0.055],
        'D': [0.303,-0.057,-0.014,0.225,0.156],
        'C': [-0.132,0.174,0.07,0.565,-0.374],
        'Q': [0.149,-0.184,0.03,0.035,-0.112],
        'E': [0.221,-0.280,-0.315,0.157,0.303],
        'G': [0.218,0.562,-0.024,0.018,0.106],
        'H': [0.023,-0.177,0.041,0.28,-0.021],
        'I': [-0.353,0.071,-0.088,-0.195,-0.107],
        'L': [-0.267,0.018,-0.265,-0.274,0.206],
        'K': [0.243,-0.339,-0.044,-0.325,-0.027],
        'M': [-0.239,-0.141,-0.155,0.321,0.077],
        'F': [-0.329,-0.023,0.072,-0.002,0.208],
        'P': [0.173,0.286,0.407,-0.215,0.384],
        'S': [0.199,0.238,-0.015,-0.068,-0.196],
        'T': [0.068,0.147,-0.015,-0.132,-0.274],
        'W': [-0.296,-0.186,0.389,0.083,0.297],
        'Y': [-0.141,-0.057,0.425,-0.096,-0.091],
        'V': [-0.274,0.136,-0.187,-0.196,-0.299]}

# calling main function for allergens.csv
main(allergen_file, allergen_output_file, allergen_label, j_list, k_list, lag)
print(f'File {allergen_output_file} has been successfully created')

# calling main function for NonAllergens.csv
```

```
main(non_allergen_file, non_allergen_output_file, non_allergen_label, j_list, k_list, lag)
print(f'File {non_allergen_output_file} has been successfully created')
```

Algorithms used for classification

Gaussian Naïve Bayes

```
from sklearn.naive_bayes import GaussianNB
nb = GaussianNB()
nb.fit(x_train, y_train)
print("Naive Bayes score: ",nb.score(x_test, y_test))
y_pred = nb.predict(x_test)
print(classification_report(y_test, y_pred))
```

Output :

```
Naive Bayes score:  0.6414027149321267
              precision    recall  f1-score   support

           0       0.60      0.83      0.70       435
           1       0.74      0.46      0.56       449

    accuracy                           0.64       884
   macro avg       0.67      0.64      0.63       884
weighted avg       0.67      0.64      0.63       884
```

Radius Neighbours Classifier

```
from sklearn.neighbors import RadiusNeighborsClassifier
from sklearn.model_selection import cross_val_score
from sklearn.model_selection import RepeatedStratifiedKFold
model = RadiusNeighborsClassifier()
model.fit(x_train, y_train)

# testing our model on the testing data
print('Accuracy: ',model.score(x_test, y_test))
y_pred = model.predict(x_test)
print(classification_report(y_test, y_pred))
```

Output :

```
Accuracy:  0.4920814479638009
              precision    recall  f1-score   support

           0       0.49      1.00      0.66       435
           1       0.00      0.00      0.00       449

    accuracy                           0.49       884
   macro avg       0.25      0.50      0.33       884
weighted avg       0.24      0.49      0.32       884
```

Extra Tree Classifier

from sklearn.ensemble import ExtraTreesClassifier

extra_tree_forest = ExtraTreesClassifier(n_estimators = 100, criterion ='entropy', max_features = 'sqrt')

extra_tree_forest.fit(x_train, y_train)

testing our model on the testing data

print('Accuracy: ',extra_tree_forest.score(x_test, y_test))

y_pred = extra_tree_forest.predict(x_test)

print(classification_report(y_test, y_pred))

Output:

```
Accuracy:  0.9015837104072398
              precision    recall  f1-score   support

           0       0.86      0.95      0.91       435
           1       0.95      0.85      0.90       449

    accuracy                           0.90       884
   macro avg       0.91      0.90      0.90       884
weighted avg       0.91      0.90      0.90       884
```

Bagging Classifier

from sklearn.ensemble import BaggingClassifier

from sklearn import model_selection

from sklearn.tree import DecisionTreeClassifier

kfold = model_selection.KFold(n_splits = 3, random_state = None)

base_cls = DecisionTreeClassifier()

num_trees = 100

model = BaggingClassifier(base_estimator = base_cls, n_estimators = num_trees, random_state = None)

results = model_selection.cross_val_score(model, x, y, cv = kfold)

print("accuracy: ",results.mean())

model.fit(x_train, y_train)

y_pred = model.predict(x_test)

print(classification_report(y_test, y_pred))

Output:

```
accuracy:  0.8583670857586411
              precision    recall  f1-score   support

           0       0.86      0.89      0.88       435
           1       0.89      0.86      0.88       449

    accuracy                           0.88       884
   macro avg       0.88      0.88      0.88       884
weighted avg       0.88      0.88      0.88       884
```

ADA Boost

from sklearn.ensemble import AdaBoostClassifier

clf = AdaBoostClassifier(n_estimators=100, base_estimator= None,learning_rate=1, random_state = 96)

clf.fit(x_train, y_train)

print('Accuracy: ',clf.score(x_test, y_test))

y_pred = clf.predict(x_test)

print(classification_report(y_test, y_pred))

Output:

```
Accuracy:  0.7692307692307693
              precision    recall  f1-score   support

           0       0.75      0.79      0.77       435
           1       0.79      0.75      0.77       449

    accuracy                           0.77       884
   macro avg       0.77      0.77      0.77       884
weighted avg       0.77      0.77      0.77       884
```

Linear Discriminant Analysis

from sklearn.discriminant_analysis import LinearDiscriminantAnalysis

model = LinearDiscriminantAnalysis()

model.fit(x_train, y_train)

print('Accuracy: ',model.score(x_test, y_test))

y_pred = model.predict(x_test)

print(classification_report(y_test, y_pred))

Output:

```
Accuracy:  0.7613122171945701
              precision    recall  f1-score   support

           0       0.73      0.81      0.77       435
           1       0.80      0.71      0.75       449

    accuracy                           0.76       884
   macro avg       0.76      0.76      0.76       884
weighted avg       0.76      0.76      0.76       884
```

Quadratic Discriminant Analysis

```
from sklearn.discriminant_analysis import QuadraticDiscriminantAnalysis
model = QuadraticDiscriminantAnalysis()
model.fit(x_train, y_train)
print('Accuracy: ',model.score(x_test, y_test))
y_pred = model.predict(x_test)
print(classification_report(y_test, y_pred))
```

Output:

```
Accuracy:  0.8427601809954751
              precision    recall  f1-score   support

           0       0.78      0.94      0.86       435
           1       0.93      0.74      0.83       449

    accuracy                           0.84       884
   macro avg       0.86      0.84      0.84       884
weighted avg       0.86      0.84      0.84       884
```

LSTM (Long short-term memory)

```
import pandas as pd
import numpy as np
from sklearn.model_selection import train_test_split
from sklearn.metrics import accuracy_score, confusion_matrix , classification_report
from keras import models
from keras import layers
from tensorflow.keras.utils import to_categorical

df = pd.read_csv("allergen_non_allergen_merged.csv")
df = df.drop(columns=['Header'])

dfx = df.iloc[:,:-1]
```

```
x=dfx.to_numpy()
dfy=df.iloc[:,-1]
y=dfy.to_numpy()
x_train,x_test,y_train,y_test=train_test_split(x,y,test_size=0.2,random_state=2)
# One hot encode output labels
train_labels = to_categorical(y_train)
test_labels = to_categorical(y_test)
# Create the network
model = models.Sequential()
model.add(layers.Dense(512, activation='relu', input_shape=(125,)))
model.add(layers.Dropout(0.20))
model.add(layers.Dense(1024, activation='relu', input_shape=(125,)))
model.add(layers.Dropout(0.40))
model.add(layers.Dense(1024, activation='relu', input_shape=(125,)))
model.add(layers.Dense(2, activation='softmax'))

# Compile the network
model.compile(optimizer='rmsprop', loss='categorical_crossentropy', metrics=['accuracy'])
# Train the network
model.fit(x_train, train_labels, epochs=100, batch_size=40)
# Make prediction
predictions=np.argmax(model.predict(x_test),axis=1)
_, test_acc = model.evaluate(x_test, test_labels)
print('Test Accuracy: ', test_acc * 100)
print(classification_report(y_test,predictions))
print("\n Accuracy score: ",accuracy_score(y_test,predictions))
```

Output:

```
Epoch 1/100
3536/3536 [==============================] - 5s 1ms/step - loss: 0.6129 - accuracy: 0.6403
Epoch 2/100
3536/3536 [==============================] - 5s 1ms/step - loss: 0.4985 - accuracy: 0.7624
Epoch 3/100
3536/3536 [==============================] - 5s 1ms/step - loss: 0.4457 - accuracy: 0.7893
Epoch 4/100
3536/3536 [==============================] - 5s 1ms/step - loss: 0.4049 - accuracy: 0.8213
Epoch 5/100
3536/3536 [==============================] - 5s 1ms/step - loss: 0.3687 - accuracy: 0.8326
Epoch 6/100
3536/3536 [==============================] - 5s 1ms/step - loss: 0.3231 - accuracy: 0.8563
Epoch 7/100
3536/3536 [==============================] - 5s 1ms/step - loss: 0.3000 - accuracy: 0.8660
Epoch 8/100
3536/3536 [==============================] - 5s 1ms/step - loss: 0.2604 - accuracy: 0.8872
Epoch 9/100
3536/3536 [==============================] - 5s 1ms/step - loss: 0.2493 - accuracy: 0.8914
Epoch 10/100
```

```
3536/3536 [==============================] - 5s 1ms/step - loss: 0.0233 - accuracy: 0.9912
Epoch 94/100
3536/3536 [==============================] - 5s 1ms/step - loss: 0.0197 - accuracy: 0.9960
Epoch 95/100
3536/3536 [==============================] - 5s 1ms/step - loss: 0.0344 - accuracy: 0.9941
Epoch 96/100
3536/3536 [==============================] - 5s 1ms/step - loss: 0.0221 - accuracy: 0.9915
Epoch 97/100
3536/3536 [==============================] - 5s 1ms/step - loss: 0.0063 - accuracy: 0.9986
Epoch 98/100
3536/3536 [==============================] - 5s 1ms/step - loss: 0.0173 - accuracy: 0.9966
Epoch 99/100
3536/3536 [==============================] - 4s 1ms/step - loss: 0.0133 - accuracy: 0.9960
Epoch 100/100
3536/3536 [==============================] - 4s 1ms/step - loss: 0.0143 - accuracy: 0.9960
884/884 [==============================] - 0s 285us/step
Test Accuracy:  91.51583909988403

 Accuracy score:  0.915158371040724
```

Fig 6. Epoch of LSTM Algorithm

When compared to the above classification methods, LSTM performed well. The model has been developed with the creation of a network with two different activation functions namely – SoftMax and relu. Rmsprop has been used as an optimizer for the model. The network has been trained with 100 epochs each with a batch size of 40.

WEB SERVER (ProAll-D)

A web server namely, ProAll-D has been developed to predict the potential allergens using the LSTM algorithm. It is developed using the Python Django framework which is fast and user-friendly. The detailed functioning of the webserver has been described in this section as mentioned in methods.

```
Anaconda Prompt (Anaconda3)

(base) C:\Users\Rakshitha>cd Desktop\allergen_web_app_new_17_03_22\allergen_web_app_new_17_03_22\all_projects\allergen_GUI

(base) C:\Users\Rakshitha\Desktop\allergen_web_app_new_17_03_22\allergen_web_app_new_17_03_22\all_projects\allergen_GUI>python manage.py runserver
```

Fig 7. Command for executing the webserver

Firstly the path of the web app folder should be copied and pasted in cmd. Then within the allergen_GUI folder, there is a python file namely “manage.py” to execute this the second command has to be followed.

Once the above commands are executed, we get the link of the local host (“http://127.0.0.1:8000/)

```
System check identified no issues (0 silenced).
March 31, 2022 - 23:25:20
Django version 2.2.5, using settings 'allergen_GUI.settings'
Starting development server at http://127.0.0.1:8000/
Quit the server with CTRL-BREAK.
```

Copy-paste the link in the browser

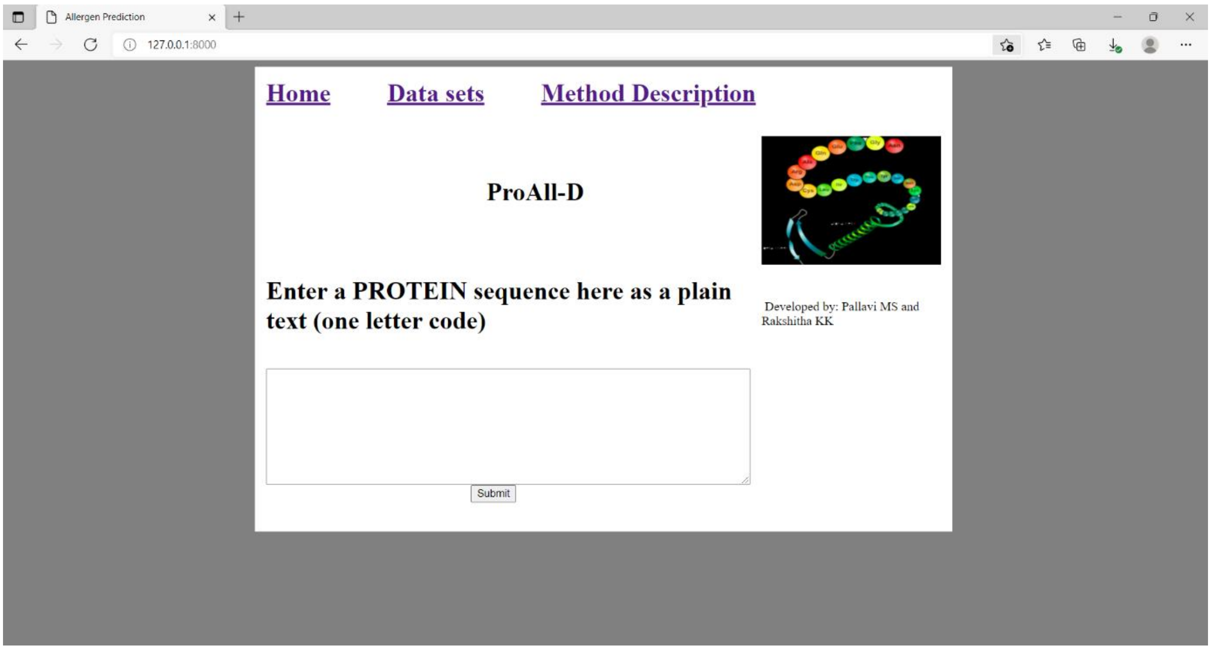

Fig 8. Interface of ProAll-D

There are three different sections namely Home, Datasets, and Method Description. In the home section, the user enters the protein sequence in a one-letter code, where the models predict whether the entered sequence is allergenic or non-allergenic. The data-set part consists of the data considered in this research in the fasta file format. The Method description provides the user with a brief description of the methodologies that we have considered.

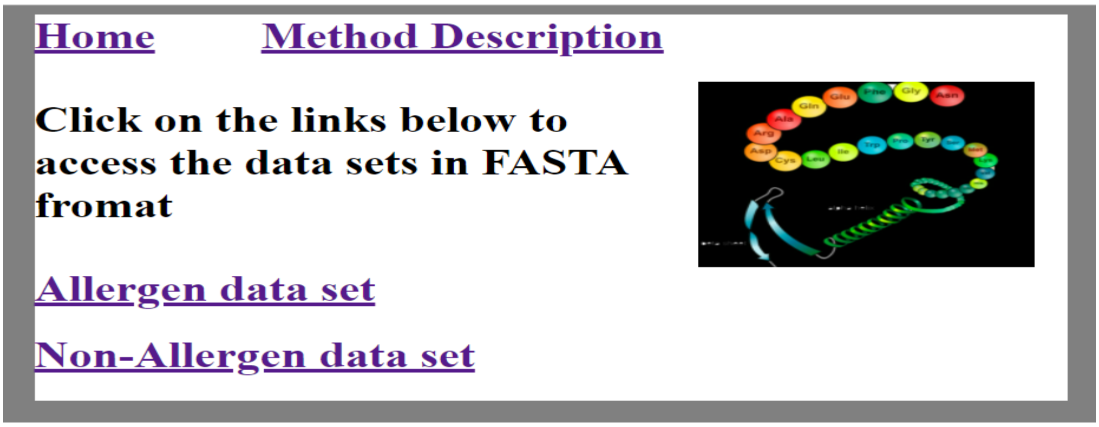

Fig 9. Dataset Section

The Dataset section consists of two links that navigate to allergen and non-allergen data, which is considered in the current research. Method Description provides a brief description of the entire process.

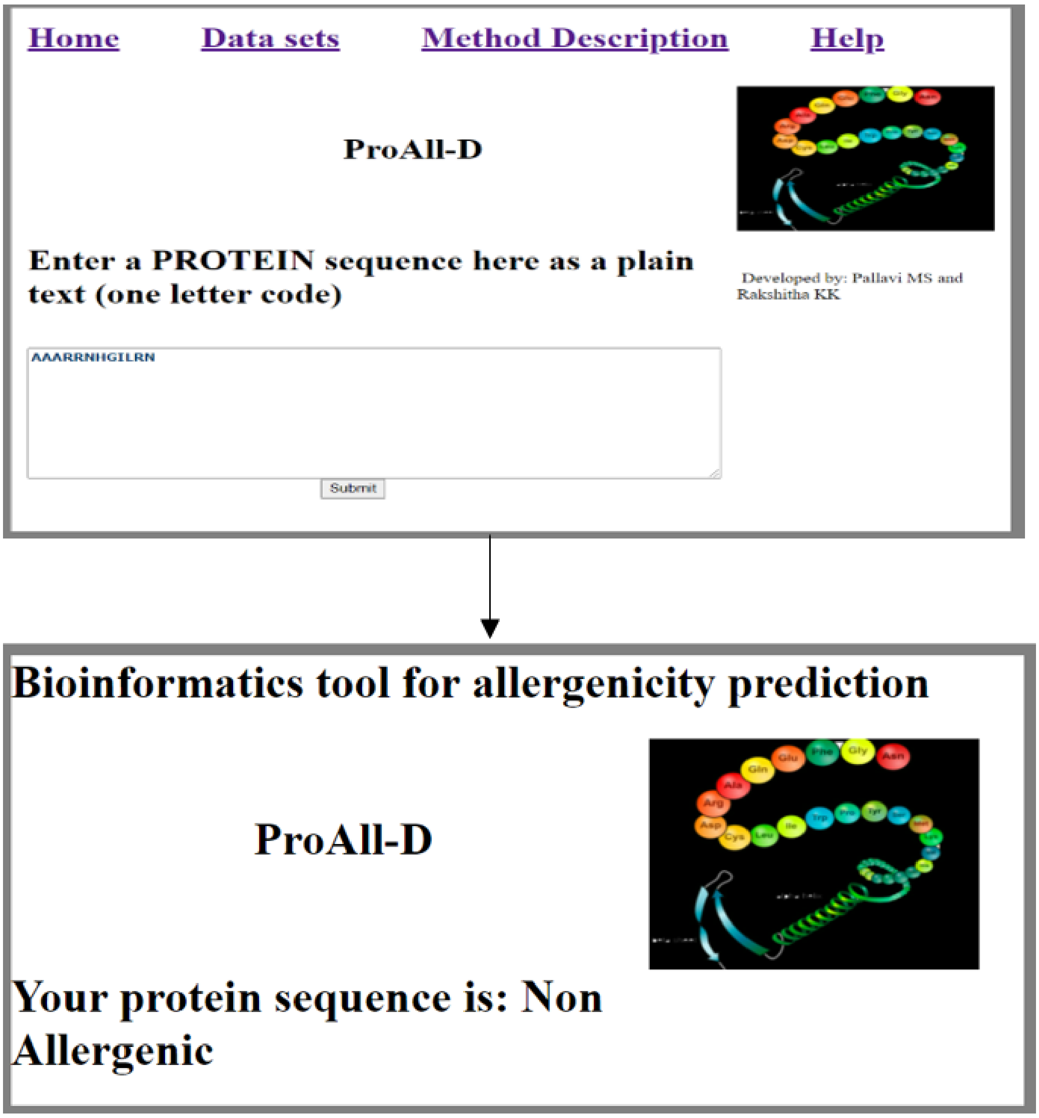

Fig 10. Working of ProAll-D

The user has to enter the protein sequence in a one-letter code in the home section. The model predicts whether the entered sequence is allergenic or non-allergenic. Suppose if the user enters a character apart from 20 naturally occurring amino acids, then we get a message as an undefined character for the entered sequence.

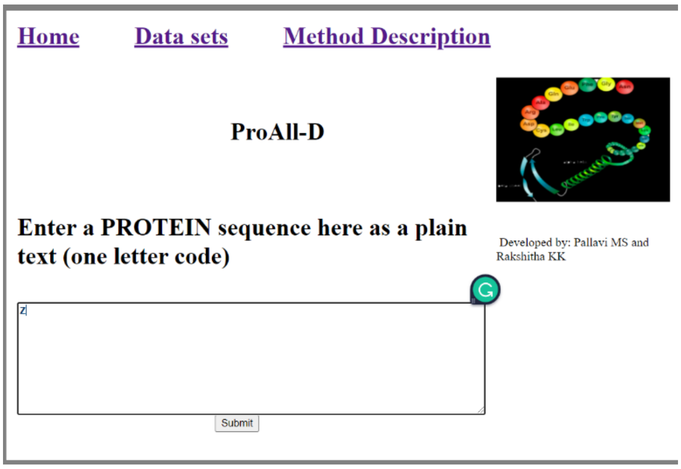

Here the entered character is “Z” which is not a part of the aminoacid character, then we get the result as:

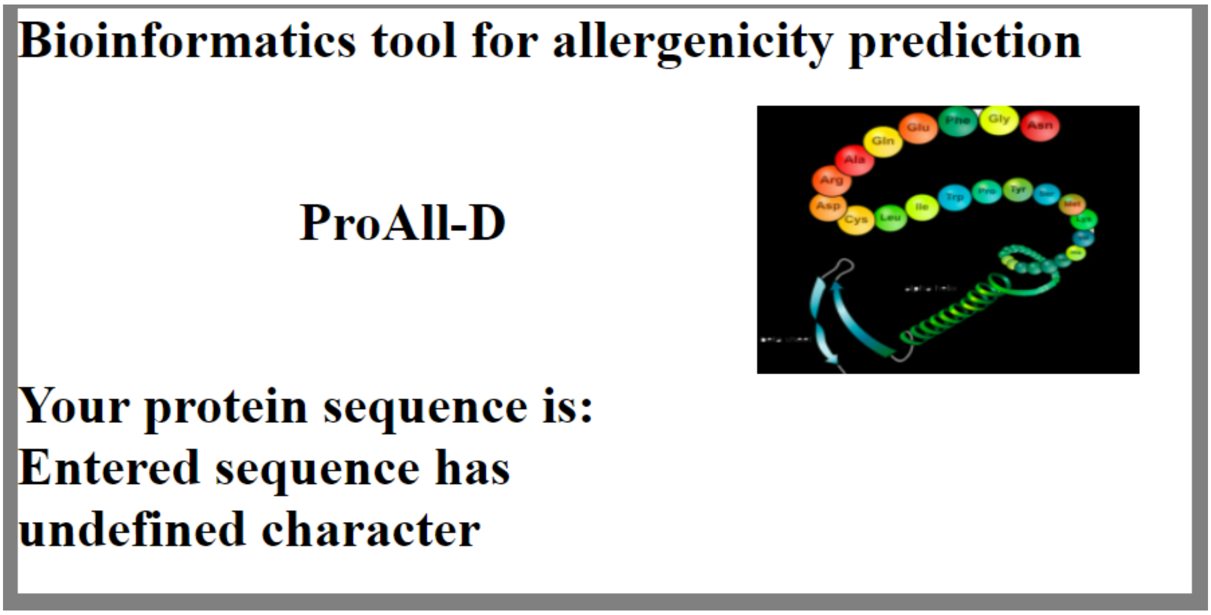

Fig 11. Checking for exceptional characters
